# Spaln3: improvement in speed and accuracy of genome mapping and spliced alignment of protein query sequences

**DOI:** 10.1093/bioinformatics/btae517

**Published:** 2024-08-17

**Authors:** Osamu Gotoh

**Affiliations:** Department of Computational Biology and Medical Sciences, Graduate School of Frontier Sciences, The University of Tokyo, Kashiwa, Chiba 277-8562, Japan; Artificial Intelligence Research Center, National Institute of Advanced Industrial Science and Technology (AIST), Koto-ku, Tokyo 135-0064, Japan

## Abstract

**Motivation:**

Spaln is the earliest practical tool for self-sufficient genome mapping and spliced alignment of protein query sequences onto a mammalian-sized eukaryotic genomic sequence. However, its computational speed has become inadequate for the analysis of rapidly growing genomic and transcript sequence data.

**Results:**

The dynamic programming calculation of Spaln has been sped up in two ways: (i) the introduction of the multi-intermediate unidirectional Hirschberg method and (ii) SIMD-based vectorization. The new version, Spaln3, is ∼7 times faster than the latest Spaln version 2, and its gene prediction accuracy is consistently higher than that of Miniprot.

**Availability and implementation:**

https://github.com/ogotoh/spaln.

## 1 Introduction

Annotating genomic sequences of nonmodel eukaryotes accumulating daily is an urgent task for extracting valuable information on the biology and evolution of organisms. Finding protein-coding genes in the genome is the first and one of the most important steps in genome annotation. Recent synthetic approaches (Holt and Yandell 2011, Brůna *et al.* 2021) have incorporated such information as the intrinsic sequence properties of the genome, the short- and long-read sequences of transcribed RNA, and the homologous amino acid sequences or CDSs of other species. RNA and cross-species information is usually utilized in spliced alignment. As amino acid sequences are more robust than CDSs against evolutionary changes, the spliced alignment of protein query sequences is preferred in various situations.

The first decade of the 2000s saw the development of several programs for the spliced alignment of protein query sequences (Iwata and Gotoh 2012). However, most of these programs relied on external “mapping” programs like Blast (Altschul *et al.* 1990) to locate the target genes in the genome. Spaln (Gotoh 2008) is the first practical tool for genome mapping and spliced alignment on a conventional personal computer, and has been continuously maintained and improved since then. Very recently, however, Li released Miniprot and reported that it is ∼30 times faster than Spaln2 and has nearly equivalent gene prediction accuracy (Li 2023).

In this report, we introduce a new version of Spaln, Spaln3. Compared with the previous version, Spaln2, Spaln3 features two major advances in dynamic programming (DP) calculation, the most time-consuming part of the entire algorithm. Spaln3 provides four calculation modes (A0–A3) depending on the use or nonuse of SIMD-based vectorization and the nonlinear intron length distribution treatment. Experiments on nine pairs of reference genomes and various sets of cross-species queries indicate that modes A0 and A1 of Spaln3 are more accurate and memory-efficient than Spaln2, whereas the fastest but least accurate mode A3 is ∼7 times faster than Spaln2 with comparable gene prediction accuracy. Although Spaln3 is still slower than Miniprot even in mode A3, all modes of Spaln3 significantly outperform Miniprot in terms of gene-level accuracy (all exon boundaries including translational initiation and termination sites are correctly identified). Whereas the exon-level sensitivity (recall) of Spaln3 is equivalent to or slightly higher than that of Miniprot, its exon-level specificity (precision) is consistently higher than that of Miniprot. The Spaln3 package affords a tool for generating a species-specific parameter set if the genomic sequence and an appreciable number of cognate transcript [cDNA, CDS, EST, or TSA (transcriptome shotgun assembly)] sequences are provided by the user.

## 2 Materials and methods

### 2.1 Multi-intermediate unidirectional Hirschberg method

Huang and Zhang were the first to apply a DP algorithm for the spliced alignment of protein query sequences (Huang and Zhang 1996). They also adopted the Hirschberg linear-space algorithm (Hirschberg 1975) to reduce the memory requirement from ordinary O(*mn*) to O(*n*), where *m* and *n* are, respectively, the lengths of the query and the genomic segment to be aligned. However, because their algorithm was complicated and time-consuming, their implementations, NAP and LAP, were not widely used. On the other hand, Spaln (Gotoh 2008) inherited the DP algorithm from its predecessor Aln (Gotoh 2000). Although the Aln/Spaln algorithm was much simpler than the NAP/LAP algorithm, implementation of the bidirectional Hirschberg (BDH) method was difficult for the following reasons: the algorithm that takes coding frames and introns into account was intrinsically complicated even for score-only calculations; the large parts of the codes for forward and backward calculations could not be shared owing to the asymmetric nature of the genomic sequence; and finding the midpoint was not easy in the presence of reading frames and introns with affine or more general gap penalties.

The idea that only the forward calculation is needed for sequence alignment in linear space came from Eppstein (unpublished), as documented by Hirschberg (Hirschberg 1997). Unfortunately, this unidirectional approach (Powell *et al.* 1999), referred to herein as the unidirectional Hirschberg (UDH) method, failed to attract as much attention as the popular BDH approach (Myers and Miller 1988), partly because it required twice as large memory as the BDH method. However, the UDH method could circumvent the second and third difficulties mentioned in the previous paragraph. Hence, since version 2.4.0, Spaln has changed its main alignment engine from the BDH method to the UDH method. The UDH method offers two additional benefits. First, under the semi-global alignment mode (terminal gaps are not penalized), the recalculation area can be reduced. Second, the UDH method can be vectorized with minimal effort in addition to the effort spent for score-only calculations.

To extend the benefits of the single-intermediate UDH (SIUDH) algorithm, we have devised a new algorithm, the multi-intermediate UDH (MIUDH) algorithm. Both the BDH method and the SIUDH method set the center row (or column) within which the midpoint is searched. The computation time for the recursive operation of this procedure is ∼2 times longer than that for the score-only calculation. However, we do not need to follow the divide-and-conquer paradigm up to the final stage. Instead, we may break off the recursion when the space necessary for 2D traceback information becomes less than a certain threshold, *V_max_* (Supplementary Fig. S1a). In addition, we can save recalculation time by setting more than one intermediate (Supplementary Fig. S1b), sometimes called a checkpoint (Grice *et al.* 1997). Although Powell *et al.* (1999) discussed a general scheme, we adapted this approach for our specific problem.

Let *k *>* *0 be the number of intermediates, and *m* and *n* be, respectively, the lengths of the query, ***a*** = *a*_0_*a*_1_…*a_i_*…*a_m_*_-1_, and the genomic segment, ***b*** = *b*_0_*b*_1_…*b_j_*…*b_n_*_-1_, sequences. Hereinafter, *i* and *j* are used without notification as the coordinates of sequences ***a*** and ***b***, respectively. For each intermediate $ii\left[c\right]=cm/\left(k+1\right),\;c\in \left[1,k\right]$, we prepare two 2D integer arrays, *VL*[*c*] and *HL*[*c*] of size d×n (thus, *VL* and *HL* are 3D arrays), where *d* denotes the number of pieces of a piecewise-linear gap penalty function (Gotoh 1990). In our case of an affine gap penalty function, d=2, where *VL*[*c*][0][*j*] and *VL*[*c*][1][*j*] correspond to the diagonal and vertical moves in sequence alignment, respectively. Each element *VL*[*c*][*p*][*j*] ($c\in \left[2,k\right]$, $p\in \left[0,d-1\right]$, $j\in \left[0,n\right]$) stores the *j*-coordinate at which the optimal path leading to the present cell on the *c*-th intermediate crosses the (*c*-1)-th intermediate, whereas *VL*[1][*p*][*j*] stores the *j*-coordinate at which the alignment starts. Whereas the values of *VL*[1][*p*][*j*] are fixed to the left end of the genomic segment under the global alignment setting, they can be increased under the semi-global setting. The coordinate information is created in the initialization process, propagated to the next intermediate along with the MAX operation at each DP cell, and stored in *VL* and refreshed at the following intermediates. On the other hand, *HL* works somewhat differently from *VL*; each element of *HL* is the *j*-coordinate on $ii\left[c\right]\;$at which the current horizontal gap starts, the nearest intron ends (potential acceptor site), or the corresponding intron starts (donor site) if the current site is a potential acceptor site, where a potential donor or acceptor site is defined as a site at which the splicing signal strength is greater than a certain threshold or a site that obeys a certain predefined rule. Because of these features, we call *VL* “vertical link” and *HL* “horizontal link” (Supplementary Fig. S1b). A detailed pseudocode of the above algorithm is shown in Supplementary Fig. S2. Although *HL* may be dispensable, it simplifies and accelerates the recalculation when introns happen to reside on some intermediates, as exemplified by the second intermediate in Supplementary Fig. S1b. *HL*[*c*][1][*j*] manipulates the rare case of a deletion in the query striding across an intron [case (e) in the inset of Supplementary Fig. S2].

If we use a four-byte integer as an element of the vertical and horizontal link arrays, the total memory for storing *k* sets of them is 4×4×k×n bytes for d=2. Assuming that we pack the traceback information into a two-byte integer at each DP cell, the approximate memory needed is $2\times m\times n\times {\left(k+1\right)}^{-2}\;$bytes. Note that the variables used in the first-phase DP calculation may be erased after the cell that gives the maximal score is identified, and the variables used in the recalculation have a negligible impact on memory usage. Hence, $\hat{k}$ that minimizes the following function

(1) $$M\left(k,m,n\right)=16nk+2mn{\left(k+1\right)}^{-2}$$

is expected to be the most economical for memory usage. Solving

(2) $$\partial M(k,m,n)/\partial k=16n-4mn{\left(\hat{k}+1\right)}^{-3}=0,$$

we obtain

(3) $$\hat{k}\left(m\right)\approx \sqrt[{3}]{m/4}-1.$$

Let $B\left(m,n\right)$ and *C* be constants independent of *k*. The time used for recalculation, $TR\left(k\right)$, is estimated as:

(4) $$TR\left(k,m,n\right)\approx \mathrm{Cmn}/\left(k+1\right)+B\left(m,n\right).$$

The above consideration is valid only if the expected memory size does not exceed the given threshold, $V_{\text{max}}.\;$Otherwise, we resort to the more space-saving recursive SIUDH algorithm. By default, MIUDH and SIUDH algorithms are automatically chosen depending on whether $M(\hat{k}\left(m\prime \right),m\prime ,n\prime )<V_{\text{max}}$ or not, where m′ and n′ are, respectively, the lengths of query and genomic segment substrings delineated by the SIUDH method. We use this hybrid approach unless otherwise specified.

### 2.2 SIMD-based vectorization

Spaln3 adopts the anti-rhombic coordinate system proposed by Li (2018) to implement the SIMD-based vectorization of the DP algorithm with a banded approximation (Supplementary Fig. S3). However, the nonlinear intron penalty as a function of intron length (Gotoh 2008) hampers straightforward implementation. To partially circumvent this problem, Spaln3 offers four calculation modes, A0–A3, which are respectively invoked by -A[0–3] options. The objective functions of all the four modes are the same as one another except for the precision in intron penalty function and rigorousness in calculating alignment score around splice junctions. The algorithmic differences among the mode A0–A3 in calculating intron penalty are illustrated in Supplementary Fig. S4. Mode A0 is most rigorous relying on only scalar operations and is thus equivalent to earlier versions in this respect. Mode A1 vectorizes most parts of the DP operations except the routines concerned with the intron penalty, which rely on scalar operations. Mode A3 uses a constant, length-independent intron penalty, as the first version of Aln (Gotoh 2000), Miniprot, and most other spliced alignment methods. Finally, mode A2 uses a coarse-grained intron penalty function (Supplementary Fig. S5) instead of the full-precision intron penalty function used by modes A0 and A1. Although modes A2 and A3 can be fully vectorized, the grain size has to be quite large as the available number of vector registers is limited. Moreover, mode A2 may fail to run owing to a shortage of vector registers on a machine equipped with CPUs of an architecture older than AVX2.

In addition to the default mixed approach, Spaln3 also supports the recursive SIUDH method devoid of the MIUHD algorithm. The recursive modes are invoked by -A[4–7] options, which are parallel to -A[0–3] options with respect to the vectorization and treatment of the intron penalty.

### 2.3 Minor changes in heuristic methods

Slight modifications were made to improve the sensitivity and specificity of the HSP (high-scoring pair) search. For example, the reduced alphabet size, the *k*-mer weight, and the bit pattern of spaced seeds of the third-level recursive HSP search were respectively changed from 12, 3, and “1101” to 10, 4, and “11011” by default. In addition, the boundaries and score of each HSP were more precisely delineated than before with the ungapped Smith and Waterman (1981) algorithm applied to each diagonal that contains more *k*-mer hits than a prespecified number.

### 2.4 Preparation of test programs and data

The genomic sequences, the gene annotation files in GFF format, and the amino acid sequences of four “reference” genomes (*Arabidopsis thaliana*, *Caenorhabditis elegans*, *Drosophila melanogaster*, and *Homo sapiens*) were downloaded from the Refseq FTP site (https://ftp.ncbi.nlm.nih.gov/genomes/refseq/) (Supplementary Table S1). The amino acid sequences of nine other species used for queries were also downloaded from the same site: *Glycine max* and *Zea mays* for *A.thaliana*; *Caenorhabditis briggsae* for *C.elegans*; *Anopheles gambiae*, *Drosophila yakuba*, and *Musca domestica* for *D.melanogaster*; and *Danio rerio*, *Gallus gallus*, and *Mus musculus* for *H.sapiens* (Supplementary Table S1). For both references and queries, we selected the amino acid sequences evidenced by mRNA (prefixed by NP_) if an appreciable number (>5000) of such “NP_” sequences were available. Otherwise, all registered amino acid sequences were subjected to the following filtering procedures. We removed splicing isoforms from the initial reference sets to reduce redundant evaluation, retaining only the longest ones. For each pair of sets of reference and query amino acid sequences, we performed an all-by-all Blast search to obtain the reciprocal best-hit pairs (Tatusov *et al.* 1977). The gene structure of each reference was obtained from the corresponding annotation and used as the gold standard. Table 1 lists some properties of the genome and query pairs used in our examination. It should be noted that the set of tested genes is a subset of the whole complement of annotated protein-coding genes in each reference genome.

**Table 1.** Properties of reference and query sequences used for examination.

Reference	Genome size (Mb)	Gene size^a^^,^^b^ (kb)	Query	Code^c^	Number^d^	Aa identity^a^ (%)	Query size^a^ (aa)
*A.thaliana* ^e^	119.7	215.1	*G.max* ^e^	AG6	2993	60.42	304.8
		219.8	*Z.mays* ^e^	AZ8	6401	53.86	429.0
*C.elegans* ^e^	100.3	231.4	*C.briggsae*	CC4	12091	72.25	456.6
*D.melanogaster* ^e^	143.7	245.0	*A.gambiae*	DA9	4855	49.69	567.7
		241.4	*D.yakuba*	DD1	10100	90.11	541.4
		246.1	*M.domestica*	DM7	6589	59.83	609.7
*H.sapiens* ^e^	3298.4	690.7	*D.rerio* ^e^	HD5	8137	62.64	491.3
		695.0	*G.gallus* ^e^	HG3	4952	74.74	499.2
		757.0	*M.musculus* ^e^	HM2	15438	84.39	595.3

a Mean of genes whose numbers are shown in the Number column.

b Each genic region spans from translational start codon minus 10 kb to stop codon plus 10 kb.

c Each code consists of the initials of reference and query species and the rank of amino acid identity.

d Number of reference genes (equals the number of queries) used for examination.

e All genes are evidence-based.

Spaln and Miniprot (https://github.com/lh3/miniprot) were respectively compiled with g++ and gcc version 9.2.0. The compiler option for Spaln includes -march=native, which enables the use of 256-bit AVX2 vector registers, whereas Miniprot appears to exclusively use the SSE4.1 instructions in our system. Reference genomic sequence *G*_g was formatted with Spaln and Miniprot in the default setting to generate *G_*g.bkp *etc* for Spaln3 and *G*_g.mpi for Miniprot. Each set of queries, *Q*, was mapped and aligned against the reference genomic sequence with the following commands:

spaln[2|3] -Q7 -T*G* -pq -A[0-3] -O0 -t24 -dG_g *Q*

and

miniprot -I -j[1|2] --gff -t24 G_g.mpi *Q*.

The -Q7 option of Spaln indicates genome mapping and alignment with heuristics, the genome identifier *G* specifies the species-specific parameter set, the -pq option suppresses warning messages, the -O0 option indicates GFF3 output, and the -t option specifies the number of threads. The -A option was omitted for Spaln2. With Miniprot, the -I option directs to estimate the maximum intron length from the genome size, the -j option specifies species-specific parameter set (-j2 for human and -j1 for the other reference genomes), and the -t option specifies the number of threads. We also tried the --out = 1 and --out = 4 options of Miniprot, but the results did not differ much from each other and from that of the default setting without the --out option. As the default setting seemed best with regard to sensitivity and speed, we show only the results of this setting in this report. All calculations were performed on a joint-use Linux-based computer system at the National Institute of Genetics (NIG) under the conditions of maximum memory < 24 GB, number of threads ≤ 24, and maximum run time < 60 min, with a few exceptions. The real elapsed time and memory usage of each run were measured with the /usr/bin/time command of the system.

For each pair of reference and query, we also prepared a multi-FASTA file, *CG*, which contains concatenated genic sequences, where each gene spans from 10 kb upstream of the translational initiation codon to 10 kb downstream of the translational termination codon. Each genic sequence in *CG* was aligned with the presumably orthologous member in *Q* in a side-by-side manner. Spaln easily accomplished this job with the -ip option as follows:

Spaln -Q[0|3] -T*G* -A[0-3] -O0 -t24 -pw -ip *CG Q*,

where the -Q0 and -Q3 options respectively indicate banded DP and heuristic anchoring with three recursive HSP search levels, and the -pw option sets the minimum alignment score to be reported to 0 (the default is 35). As Miniprot does not seem to have an equivalent option, we wrote an *ad hoc* (not necessarily highly efficient) script that performs a desired job with a single command. Instead of the -I option, -G 200K, 48K, 40K, and 40K options were used for references of *H.sapiens*, *D.melanogaster*, *A.thaliana*, and *C.elegans*, respectively.

## 3 Results

### 3.1 Optimizing computational speed and memory usage in DP calculation with combined SIUDH and MIUDH algorithms

**Figure 1.**
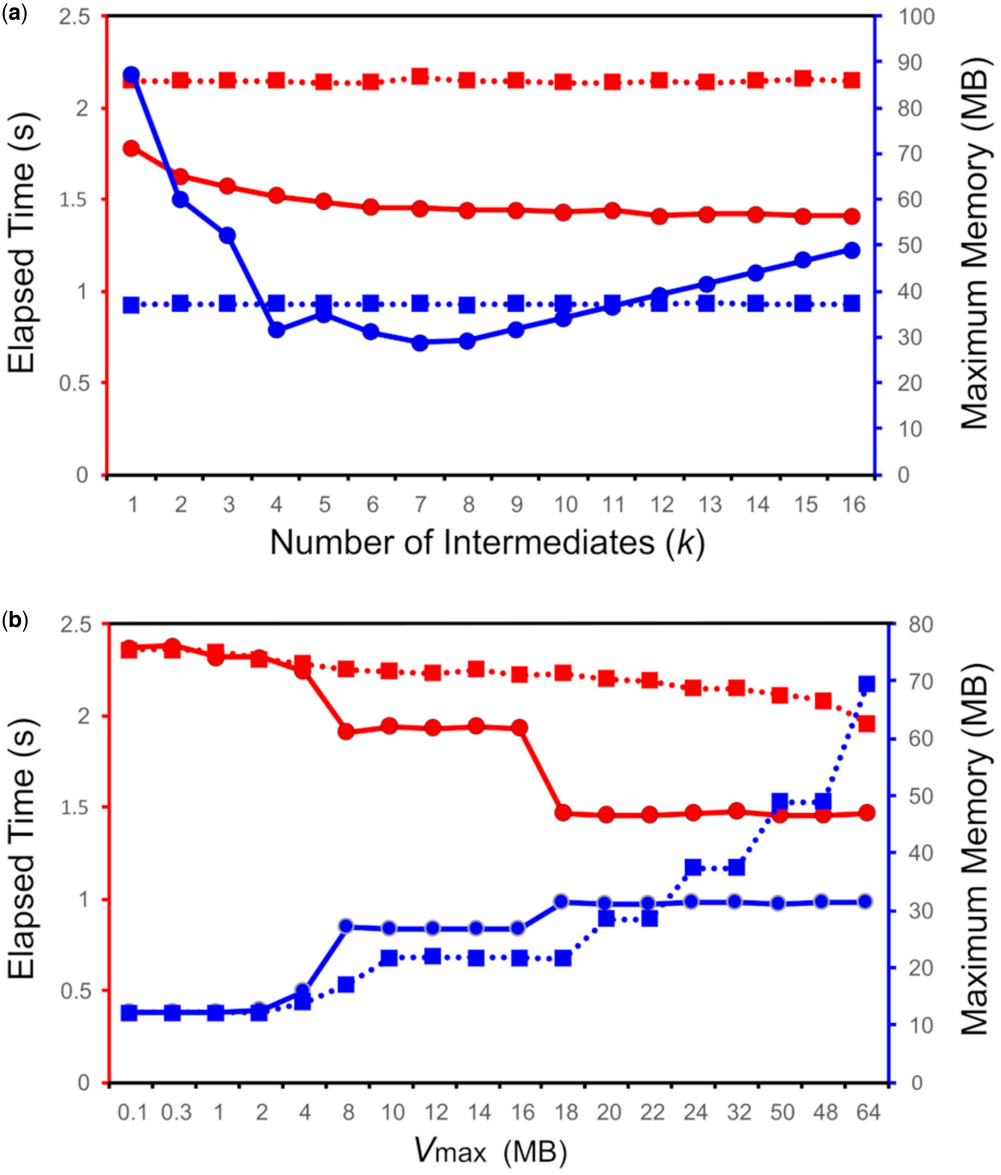
Time and memory for DP calculation with the MIUDH method (solid lines with filled circles) and the recursive SIUDH method (dotted lines with filled squares). (a) As a function of the number of intermediates. (b) As a function of the upper limit of memory ($V_{\text{max}}$) used for DP calculation. Actual memory usage is larger than this limit owing to the storage overhead for sequences and other variables.

Figure 1a demonstrates how computation time and memory usage of DP calculation in mode A3 vary with the number of intermediates *k* in the MIUDH algorithm. As a typical example, we chose a 1000-residue-long zebrafish amino acid sequence (Refseq code: NP_001112366.1) and a 159 555-bp-long human gene that codes for Refseq code: NP_003167.2 presumably orthologous to the zebrafish sequence. As expected from Equations (1)–(4), the computation time monotonically decreased with increasing *k*, whereas the memory usage had a minimum near $\hat{k}\approx \sqrt[{3}]{1000/4}-1\approx 5.3$. For this example, the MIUDH method with *k *=* *6 improved computational speed and memory usage by 31% and 33%, respectively, compared with the conventional recursive SIUDH algorithm.

To prevent the uncontrolled increase in memory usage in DP calculation, we set threshold $V_{\text{max}}$. As shown in Fig. 1b, $V_{\text{max}}$ markedly affected computation time and space even though it was fixed to 32 MB in the above experiment. This is a typical example of space–speed compensation. As expected, computation time decreased as space increased with increasing $V_{\text{max}}$. Although computation time gradually changed in the recursive SIUDH algorithm, it abruptly changed near $V_{\text{max}}$=18 MB with the default strategy, with which the recursive SIUDH algorithm and the MIUDH algorithm were automatically chosen for optimal computational speed under the given space limitation (Methods). Note that $V_{\text{max}}\;$= 18 MB is the expected value above which the default strategy uses only the MIUDH algorithm, resulting in almost constant time and memory usage independently of the explicit value of $V_{\text{max}}$.

### 3.2 Evaluation of mapping and alignment performance

We compared the performance of the three aligners, Spaln3 (version 3.0.4), Miniprot (version 0.12-r237), and the latest stable version of Spaln2 (version 2.4.13f). Other existing tools, such as GenomeThreader (Gremme *et al.* 2005) and GeMoMa (Keilwagen *et al.* 2016), were not examined as they were unlikely to outperform the three aligners (Li 2023). We evaluated the performance of the three aligners at three levels. Mapping sensitivity (*MS*) is defined as the fraction of queries for which the predicted genic area (from the translational initiation site to the termination site) overlaps at least partly with the annotated gene area. Gene-level sensitivity (*GN*) and gene-level specificity (*GP*) are respectively defined as *TP*/(*TP*+*FN*) and *TP*/(*TP*+*FP*), where *TP* stands for the number of correctly predicted genes (all predicted coding exon boundaries are identical to annotated ones), *TP*+*FN* is the total number of tested genes (which is equal to the number of queries because we assume a one-to-one correspondence between operationally identified orthologs), and *TP*+*FP* is the total number of predicted genes. Finally, exon-level sensitivity (*EN*), exon-level specificity (*EP*), and exon-level F1 measure (*EF*) were determined as described previously (Gotoh 2008).

**Figure 2.**
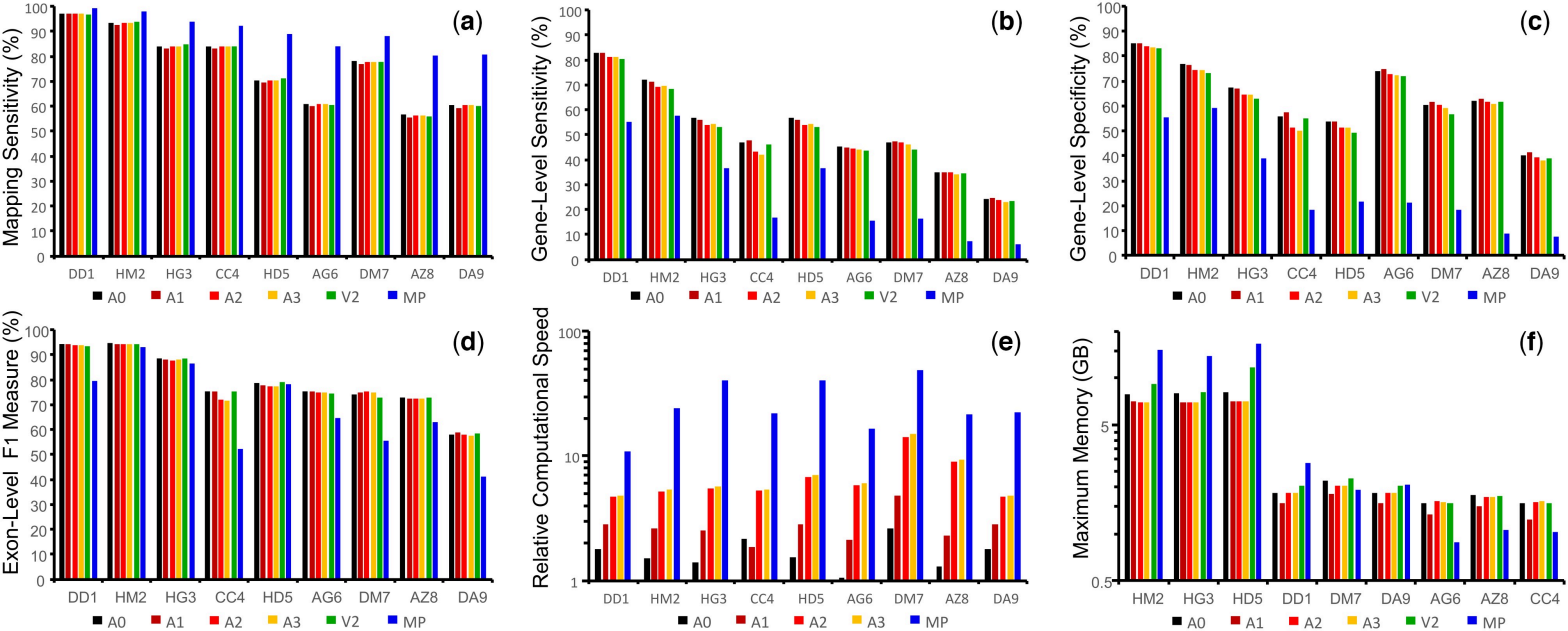
Comparison of Spaln2 (V2), Spaln3 (A0–A3), and Miniprot (MP) performance for mapping and alignment. (a) Mapping sensitivity. (b) Gene-level sensitivity. (c) Gene-level specificity. (d) Exon-level F1 measure. (e) Computational speed relative to Spaln2 in the logarithmic scale. (f) Maximum resident memory in the logarithmic scale. Genome and query pairs are represented by three-letter codes and arranged in the descending order of mean amino acid identifiers (a–e) or the descending order of genome size (f), as shown in Table 1.

As shown in Fig. 2a, the *MS*s of Spaln2/3 are inferior to those of Miniprot. However, Spaln2/3 consistently outperformed Miniprot in terms of *GN* (Fig. 2b) and *GP* (Fig. 2c). Somewhat unexpectedly, the *EN*s of Spaln2/3 are comparable to or even higher than those of Miniprot, whereas the *EP*s of Spaln2/3 are consistently higher than those of Miniprot (Supplementary Table S2). As a result, the *EF*s of Spaln3 are superior to those of Miniprot for most genome versus query pairs (Fig. 2d and Supplementary Table S2).

There are only slight differences in *MS* (Fig. 2a) and *EF* (Fig. 2d) among Spaln2 (V2) and various modes of Spaln3 (A0–A3). On the other hand, the *GNs/GPs* of modes A0–A3 show a general trend of A0 > A1 > A2 ≥A3 (Fig. 2b and c). The *GN*s/*GP*s of modes A0/A1 are consistently higher than those of V2, although some *GN*s/*GP*s of modes A2/A3 are slightly lower than those of V2.

Figure 2e shows the computational speeds of modes A0–A3 and Miniprot relative to that of Spaln2 in the logarithmic scale. With Miniprot included and Spaln2 excluded, the order of *GN*/*GP* is the inverse of that of the computational speed. The observation that all values are positive implies that modes A0–A3 of Spaln3 and Miniprot run faster than Spaln2; the mean relative computational speeds of modes A0–A3 and Miniprot are, respectively, 1.7, 2.7, 6.8, 7.1, and 27.3 times higher than that of Spaln 2. Modes A0–A3 of Spaln3 run 16.7, 10.0, 4.2, and 4.0 times slower than Miniprot. Nevertheless, the memory required by Spaln3 is approximately half that required by Miniprot for the human genome (Fig. 2f, Supplementary Table S2). In addition, Spaln3 uses less memory than Spaln2 in almost all cases examined. The relative superiority in memory usage tends to vary among Spaln3 and Miniprot for small genomes, but this is not a serious matter in most computational environments. The small memory requirement for a large genome is an advantage of Spaln3 because the program can be run on a conventional personal computer, rendering it available for routine use.

We also examined the performance of Spaln3 and Miniprot under the condition that the genic region is known within a ±10 kb margin. We tested the banded DP algorithm (Q0) of Spaln3 in addition to the default heuristic algorithm (Q3) with three recursive HSP search levels. The results shown in Supplementary Fig. S6 are similar to but more discriminative than those observed in the mapping and alignment tests (Fig. 2). The *EF*s of Spaln3 do not largely vary among the modes and are superior to those of Miniprot. At the gene level, the banded DP algorithm generally elicits higher prediction accuracies than the heuristic algorithm, most noticeably in modes A0/A1 and for distant genome versus query pairs (Supplementary Fig. S6). On the other hand, as the banded DP algorithm is one to two orders of magnitude slower than the heuristic algorithm (Supplementary Table S3), its practical advantage may be limited. However, it provides a basis for gene prediction accuracy based on spliced alignment. The present results suggest the gene prediction accuracies of heuristic methods need to be improved.

**Figure 3.**
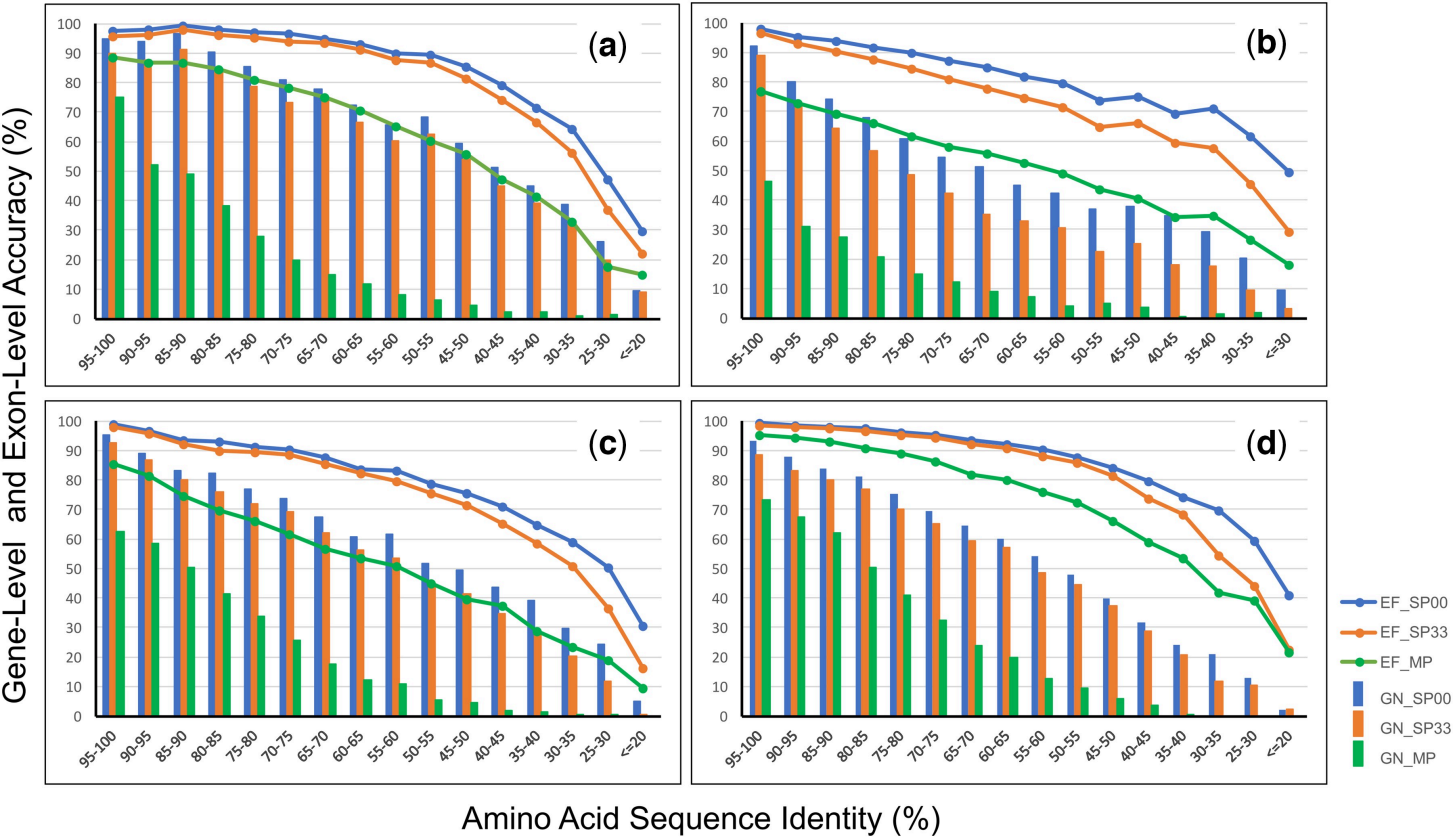
Gene-level sensitivity (GN, bar graph) and exon-level F1 measure (EF, line graph) as a function of percentage identity (*pid*) between reference and query amino acid sequences. Spaln3 with mode -Q0 -A0 (SP00), Spaln3 with mode -Q3 -A3 (SP33), and Miniprot (MP) are used. For each of the four references, *A.thaliana* (a), *C.elegans* (b), *D.melanogaster* (c), and *H.sapiens* (d), the results for all queries are pooled and rearranged into 5% sequence identity, which is defined as *pid*=100×#matches/(#matches+#mismatches+(#gaps+#unpaired residues)/2).

Figure 3 shows *GN* and *EF* as a function of percentage identity (*pid*) between query and reference amino acid sequences. For each reference genome, the results of various queries were pooled and rearranged into 5% bins of *pid*. For clarity, only the results of Miniprot, the most accurate mode -Q0-A0 of Spaln3, and the fastest but least accurate mode -Q3 -A3 of Spaln3 are shown. As expected, both *GN* and *EF* of all methods monotonically decreased with decreasing *pid*. The rates of decrease indicate marked dependence on the alignment method; Miniprot is the most susceptible and mode -Q0 -A0 of Spaln3 is the least susceptible to sequence divergence, with mode -Q3 -A3 of Spaln3 lying in between. This trend is consistent with gross statistics shown in Fig. 2 and Supplementary Fig. S6. Figure 3 also indicates that the decreasing rates are moderately dependent on the reference genome, e.g. the initial decreasing rate of *A.thaliana* (Fig. 3a) is lower than those of the other references. Several factors including average gene size, average number of exons per gene, strengths of exon/intron-recognition signals, presence of paralogs including pseudogenes and gene fragments, quality of genome assembly, and potential quality of query sequences (not all the queries are evidence-based) may influence the results. At present, however, it is difficult to quantify the relative contributions of these factors to the ease/difficulty of gene prediction.

### 3.3 Preparation of species-specific parameter sets

One potential reason why Spaln3 has higher gene prediction accuracy than Miniprot is that it uses pre-trained species-specific parameter set for each tested reference genome while Miniprot offers only two parameter sets. All experiments described in this report were performed with the parameter sets distributed in the Spaln2 package. We obtained 1211 new parameter sets by applying the script mentioned in Methods to the cognate pairs of WGS and TSA made available from the NIG database in August 2023. Of the 1035 parameter sets derived from >5000 nonredundant introns for each species, ∼90% were likely to be reliable as judged from the fraction (>98%) of the canonical intron boundary sequences, GT.AG, GC.AG, or AT.AC. After further scrutiny, these parameter sets will be added to future Spaln packages. We expect that the parameter sets of nearly 2000 species will be available soon.

## 4 Discussion

We did not examine the mapping and alignment performance of the banded DP algorithm (-Q4 option) owing to limitations in available computational resources. However, banded DP calculation may be feasible under a high-performance computational environment, especially for small or medium-sized genomes. Spaln3 offers as many as eight calculation modes (banded DP or heuristic ×A0–A3). Our results in Supplementary Fig. S6 and Supplementary Table S3 suggest a tradeoff between computational speed and prediction accuracy for the eight calculation modes and Miniprot. There may be situations where computational speed is essential for analyzing a huge amount of data. However, prediction accuracy may be more important in many practical situations. Thus, users can now choose the computational tool that best fits their data analysis needs from a variety of options.

Some queries not evidenced by mRNA might be products of misprediction. However, there were no clear signs that the fraction of nonevidenced queries significantly affected our results. Unfortunately, we could not find good test data for fungi and protists. We hope that the observations presented in this report might be extrapolated to these less explored organisms to improve the gene annotation of their genomes.

In this report, we focused on protein queries, but all the new features of Spaln3 described herein are also applicable to DNA queries. It remains to be seen how Spaln3 ranks with existing long-read aligners. One long-standing problem is how to unify the functionality of Spaln with that of Aln, which accepts multiple sequence alignments or profiles as queries. It is also worth examining whether the prediction accuracy of Spaln can be improved further by incorporating the genomic sequence features derived from deep learning algorithms (Stiehler *et al.* 2020).

## Supplementary Material

btae517_Supplementary_Data
