# Supplementary material for: Spaln3: improvement in speed and accuracy of genome mapping and spliced alignment of protein query sequences
Source: Bioinformatics. 2024 Aug 17;40(8):btae517. doi: 10.1093/bioinformatics/btae517 (PMC11361809; doi:10.1093/bioinformatics/btae517)
Supplement: btae517_Supplementary_Data [file btae517_supplementary_data.docx]

**Supplementary Material**

**Table S1. Refseq identifiers for which data were downloaded**

| Species | Refseq Identifier |
| --- | --- |
| *A. thaliana* | GCF_000001735.4_TAIR10.1 |
| *C. briggsae* | GCF_000004555.2_C |
| *C. elegans* | GCF_000002985.6_WBcel235 |
| *D. rerio* | GCF_000002035.6_GRCz11 |
| *D. melanogaster* | GCF_000001215.4_Release_6_plus_ISO1_MT |
| *D. yakuba* | GCF_016746365.2_Prin_Dyak_Tai18E2_2.1 |
| *G. gallus* | GCF_016699485.2_bGalGal1.mat.broiler.GRCg7b |
| *G. max* | GCF_000004515.6_Glycine_max_v4.0 |
| *H. sapiens* | GCF_000001405.40_GRCh38.p14 |
| *M. musculus* | GCF_000001635.27_GRCm39 |
| *M. domestica* | GCF_030504385.1_Musca_domestica.polishedcontigs.V.1.1 |
| *Z. mays* | GCF_902167145.1_Zm-B73-REFERENCE-NAM-5.0 |

**Table S2. Mapping and alignment performance comparison of Spaln2, Spaln3, and Miniprot**

| ***D. melanogaster* vs. *D. yakuba* (DD1)** | | | | | | | | |
| --- | --- | --- | --- | --- | --- | --- | --- | --- |
| Method | MS (%) | GN (%) | GP (%) | EN (%) | EP (%) | EF (%) | WCT (s) | MEM (MB) |
| A0 | 97.129 | 82.733 | 85.178 | 93.973 | 94.425 | 94.199 | 99.49 | 1827 |
| A1 | 97.000 | 82.545 | 85.097 | 93.869 | 94.499 | 94.183 | 62.62 | 1577 |
| A2 | 97.089 | 81.267 | 83.704 | 93.159 | 94.462 | 93.806 | 37.50 | 1833 |
| A3 | 97.079 | 81.129 | 83.570 | 93.132 | 94.330 | 93.727 | 36.83 | 1826 |
| V2 | 96.851 | 80.376 | 82.989 | 93.062 | 93.526 | 93.293 | 176.77 | 2048 |
| MP | 99.327 | 55.168 | 55.542 | 75.505 | 83.999 | 79.526 | 16.36 | 2843 |
| ***H. sapiens* vs. *M. musculus* (HM2)** | | | | | | | | |
| Method | MS (%) | GN (%) | GP (%) | EN (%) | EP (%) | EF (%) | WCT (s) | MEM (MB) |
| A0 | 93.451 | 71.881 | 76.918 | 93.146 | 96.387 | 94.739 | 1248.16 | 7907 |
| A1 | 92.784 | 71.058 | 76.585 | 92.444 | 96.406 | 94.384 | 724.68 | 7042 |
| A2 | 93.432 | 69.309 | 74.182 | 92.011 | 96.515 | 94.209 | 366.16 | 7012 |
| A3 | 93.438 | 69.633 | 74.523 | 92.210 | 96.507 | 94.310 | 355.58 | 6998 |
| V2 | 93.775 | 68.396 | 72.936 | 93.186 | 95.476 | 94.317 | 1895.17 | 9147 |
| MP | 97.973 | 57.747 | 58.942 | 91.385 | 94.708 | 93.017 | 79.18 | 15256 |
| ***H. sapiens* vs. *G. gallus* (HG3)** | | | | | | | | |
| Method | MS (%) | GN (%) | GP (%) | EN (%) | EP (%) | EF (%) | WCT (s) | MEM (MB) |
| A0 | 84.047 | 56.603 | 67.347 | 83.950 | 93.999 | 88.690 | 731.45 | 7993 |
| A1 | 83.219 | 55.735 | 66.974 | 83.041 | 94.053 | 88.205 | 411.72 | 6955 |
| A2 | 83.926 | 53.958 | 64.293 | 82.048 | 94.554 | 87.859 | 188.24 | 6999 |
| A3 | 83.946 | 54.180 | 64.542 | 82.308 | 94.461 | 87.967 | 181.81 | 7001 |
| V2 | 84.693 | 53.090 | 62.685 | 84.874 | 92.758 | 88.641 | 1029.94 | 8123 |
| MP | 93.881 | 36.571 | 38.955 | 82.689 | 90.146 | 86.257 | 25.72 | 13913 |
| ***C. elegans* vs. *C. briggsae* (CC4)** | | | | | | | | |
| Method | MS (%) | GN (%) | GP (%) | EN (%) | EP (%) | EF (%) | WCT (s) | MEM (MB) |
| A0 | 84.00 | 46.94 | 55.89 | 75.18 | 87.24 | 80.76 | 72.21 | 1522 |
| A1 | 83.16 | 47.87 | 57.56 | 75.44 | 88.01 | 81.24 | 43.42 | 1236 |
| A2 | 83.99 | 42.96 | 51.15 | 72.04 | 86.53 | 78.62 | 20.10 | 1606 |
| A3 | 83.97 | 42.05 | 50.07 | 71.53 | 86.07 | 78.13 | 19.87 | 1629 |
| V2 | 83.95 | 46.23 | 55.07 | 75.41 | 86.40 | 80.53 | 156.11 | 1564 |
| MP | 92.39 | 16.71 | 18.08 | 52.14 | 68.38 | 59.16 | 8.14 | 1027 |
| ***H. sapiens* vs. *D. rerio* (HD5)** | | | | | | | | |
| Method | MS (%) | GN (%) | GP (%) | EN (%) | EP (%) | EF (%) | WCT (s) | MEM (MB) |
| A0 | 70.259 | 37.778 | 53.769 | 69.135 | 90.792 | 78.497 | 1363.12 | 8167 |
| A1 | 69.362 | 37.127 | 53.526 | 68.104 | 90.884 | 77.862 | 730.99 | 7066 |
| A2 | 70.173 | 35.922 | 51.191 | 66.698 | 91.840 | 77.275 | 305.67 | 7035 |
| A3 | 70.173 | 36.082 | 51.419 | 67.063 | 91.774 | 77.496 | 293.98 | 7035 |
| V2 | 71.009 | 34.902 | 49.152 | 70.605 | 89.231 | 78.833 | 2079.24 | 11707 |
| MP | 88.730 | 19.049 | 21.468 | 72.478 | 84.774 | 78.145 | 51.90 | 16743 |
| ***A. thaliana* vs. *G. max* (AG6)** | | | | | | | | |
| Method | MS (%) | GN (%) | GP (%) | EN (%) | EP (%) | EF (%) | WCT (s) | MEM (MB) |
| A0 | 60.909 | 45.072 | 73.999 | 64.130 | 90.713 | 75.139 | 50.72 | 1567 |
| A1 | 59.973 | 44.805 | 74.708 | 63.705 | 91.569 | 75.137 | 25.33 | 1335 |
| A2 | 60.875 | 44.270 | 72.722 | 63.013 | 92.423 | 74.936 | 9.18 | 1608 |
| A3 | 60.909 | 44.036 | 72.298 | 62.856 | 92.065 | 74.707 | 8.86 | 1587 |
| V2 | 60.374 | 43.435 | 71.942 | 63.304 | 89.985 | 74.323 | 53.63 | 1567 |
| MP | 84.096 | 17.608 | 20.938 | 58.979 | 71.766 | 64.748 | 3.25 | 882 |
| ***D. melanogaster* vs. *M. domestica* (DM7)** | | | | | | | | |
| Method | MS (%) | GN (%) | GP (%) | EN (%) | EP (%) | EF (%) | WCT (s) | MEM (MB) |
| A0 | 76.400 | 41.903 | 54.847 | 68.728 | 80.453 | 74.130 | 303.91 | 2194 |
| A1 | 75.429 | 42.450 | 56.278 | 69.391 | 81.699 | 75.044 | 165.84 | 1789 |
| A2 | 76.385 | 41.994 | 54.977 | 68.108 | 84.370 | 75.372 | 55.40 | 2033 |
| A3 | 76.415 | 41.296 | 54.042 | 67.924 | 83.228 | 74.802 | 52.88 | 2037 |
| V2 | 76.172 | 39.627 | 52.022 | 69.313 | 77.035 | 72.970 | 792.44 | 2274 |
| MP | 90.090 | 14.342 | 15.920 | 50.234 | 62.211 | 55.585 | 8.58 | 1910 |
| ***A. thaliana* vs. *Z. mays* (AZ8)** | | | | | | | | |
| Method | MS (%) | GN (%) | GP (%) | EN (%) | EP (%) | EF (%) | WCT (s) | MEM (MB) |
| A0 | 56.554 | 35.010 | 61.906 | 61.865 | 88.924 | 72.967 | 138.06 | 1774 |
| A1 | 55.413 | 34.776 | 62.757 | 60.688 | 89.791 | 72.425 | 77.43 | 1510 |
| A2 | 56.460 | 34.791 | 61.621 | 60.222 | 91.163 | 72.530 | 19.94 | 1719 |
| A3 | 56.413 | 34.229 | 60.676 | 60.049 | 90.633 | 72.237 | 19.03 | 1719 |
| V2 | 56.132 | 34.541 | 61.536 | 61.623 | 88.966 | 72.813 | 178.30 | 1751 |
| MP | 80.284 | 7.140 | 8.893 | 56.422 | 70.716 | 62.765 | 8.29 | 1059 |
| ***D. melanogaster* vs. *A. gambiae* (DA9)** | | | | | | | | |
| Method | MS (%) | GN (%) | GP (%) | EN (%) | EP (%) | EF (%) | WCT (s) | MEM (MB) |
| A0 | 60.309 | 24.140 | 40.027 | 48.663 | 72.064 | 58.096 | 221.69 | 2061 |
| A1 | 59.073 | 24.408 | 41.318 | 48.812 | 73.888 | 58.788 | 120.83 | 1779 |
| A2 | 60.288 | 23.749 | 39.392 | 46.818 | 76.039 | 57.954 | 37.69 | 2044 |
| A3 | 60.309 | 23.048 | 38.217 | 46.718 | 74.530 | 57.434 | 36.00 | 2027 |
| V2 | 60.103 | 23.502 | 39.102 | 50.570 | 69.125 | 58.409 | 727.00 | 2142 |
| MP | 80.803 | 6.179 | 7.647 | 34.547 | 50.434 | 41.006 | 7.88 | 2074 |

A0–3: Calculation mode of Spaln3

V2: Spaln2

MP: Miniprot

MS: Mapping sensitivity

GN: Gene-level sensitivity

GP: Gene-level specificity

EN: Exon-level sensitivity

EP: Exon-level specificity

EF: Exon-level F1 measure

WCT: Wall clock time

MEM: Maximum resident memory

**Table S3. Spliced alignment performance comparison of Spaln2, Spaln3, and Miniprot**

| ***D. melanogaster* vs. *D. yakuba* (DD1)** | | | | | | | |
| --- | --- | --- | --- | --- | --- | --- | --- |
| Method | GN (%) | GP (%) | EN (%) | EP (%) | EF (%) | WCT (s) | MEM (MB) |
| Q0A0 | 87.000 | 87.147 | 95.853 | 95.013 | 95.431 | 718.60 | 2019 |
| Q0A1 | 86.545 | 86.665 | 95.562 | 94.924 | 95.242 | 374.15 | 776 |
| Q0A2 | 82.020 | 82.183 | 93.065 | 94.481 | 93.767 | 95.83 | 878 |
| Q0A3 | 81.921 | 82.026 | 93.197 | 94.300 | 93.745 | 87.80 | 877 |
| Q3A0 | 85.743 | 85.870 | 95.278 | 94.700 | 94.988 | 7.02 | 226 |
| Q3A1 | 85.683 | 85.811 | 95.238 | 94.754 | 94.996 | 5.57 | 192 |
| Q3A2 | 84.475 | 84.593 | 94.496 | 94.857 | 94.676 | 4.45 | 169 |
| Q3A3 | 84.396 | 84.513 | 94.494 | 94.793 | 94.643 | 4.52 | 164 |
| MP | 55.475 | 55.796 | 75.871 | 84.080 | 79.765 | 78.98 | 876 |
| ***H. sapiens* vs. *M. musculus* (HM2)** | | | | | | | |
| Method | GN (%) | GP (%) | EN (%) | EP (%) | EF (%) | WCT (s) | MEM (MB) |
| Q0A0 | 79.596 | 79.647 | 97.086 | 96.659 | 96.872 | 4450.00 | 5123 |
| Q0A1 | 78.741 | 78.792 | 96.823 | 96.665 | 96.744 | 2347.75 | 3207 |
| Q0A2 | 74.245 | 74.293 | 94.884 | 96.819 | 95.841 | 541.16 | 1714 |
| Q0A3 | 75.742 | 75.791 | 95.420 | 96.988 | 96.197 | 521.29 | 1691 |
| Q3A0 | 77.724 | 77.779 | 96.568 | 96.525 | 96.546 | 128.52 | 1166 |
| Q3A1 | 76.668 | 76.723 | 96.210 | 96.496 | 96.353 | 51.72 | 1034 |
| Q3A2 | 75.211 | 75.264 | 95.249 | 96.814 | 96.025 | 22.79 | 1218 |
| Q3A3 | 75.826 | 75.880 | 95.498 | 96.884 | 96.186 | 22.60 | 1159 |
| MP | 56.808 | 57.694 | 89.151 | 92.396 | 90.745 | 104.03 | 1495 |
| ***H. sapiens* vs. *G. gallus* (HG3)** | | | | | | | |
| Method | GN (%) | GP (%) | EN (%) | EP (%) | EF (%) | WCT (s) | MEM (MB) |
| Q0A0 | 71.062 | 71.379 | 94.974 | 94.476 | 94.724 | 2012.14 | 4075 |
| Q0A1 | 69.750 | 70.061 | 94.552 | 94.445 | 94.498 | 1048.92 | 2703 |
| Q0A2 | 65.630 | 65.923 | 91.178 | 94.993 | 93.046 | 247.05 | 1348 |
| Q0A3 | 67.407 | 67.694 | 92.039 | 95.265 | 93.624 | 238.51 | 1343 |
| Q3A0 | 68.417 | 68.764 | 93.895 | 94.223 | 94.058 | 21.67 | 1043 |
| Q3A1 | 67.084 | 67.411 | 93.417 | 94.195 | 93.804 | 13.52 | 875 |
| Q3A2 | 65.711 | 66.058 | 91.299 | 94.973 | 93.100 | 6.13 | 911 |
| Q3A3 | 66.620 | 66.971 | 91.754 | 95.094 | 93.394 | 6.04 | 865 |
| MP | 36.228 | 38.203 | 80.983 | 87.477 | 84.105 | 32.86 | 896 |
| ***C. elegans* vs. *C. briggsae* (CC4)** | | | | | | | |
| Method | GN (%) | GP (%) | EN (%) | EP (%) | EF (%) | WCT (s) | MEM (MB) |
| Q0A0 | 57.133 | 58.085 | 85.732 | 86.760 | 86.243 | 589.72 | 1663 |
| Q0A1 | 56.389 | 57.236 | 85.029 | 86.647 | 85.830 | 301.35 | 598 |
| Q0A2 | 47.093 | 47.933 | 78.031 | 85.283 | 81.496 | 80.81 | 835 |
| Q0A3 | 46.084 | 46.788 | 77.649 | 84.603 | 80.977 | 73.53 | 839 |
| Q3A0 | 52.684 | 53.764 | 81.705 | 86.128 | 83.858 | 20.91 | 91 |
| Q3A1 | 53.627 | 54.727 | 82.456 | 86.757 | 84.552 | 13.13 | 76 |
| Q3A2 | 47.804 | 48.760 | 77.596 | 85.410 | 81.316 | 6.63 | 238 |
| Q3A3 | 46.762 | 47.669 | 77.044 | 84.863 | 80.764 | 6.38 | 236 |
| MP | 15.822 | 16.961 | 50.669 | 67.607 | 57.925 | 88.20 | 163 |
| ***H. sapiens* vs. *D. rerio* (HD5)** | | | | | | | |
| Method | GN (%) | GP (%) | EN (%) | EP (%) | EF (%) | WCT (s) | MEM (MB) |
| Q0A0 | 55.745 | 56.097 | 91.056 | 89.936 | 90.493 | 1976.28 | 2019 |
| Q0A1 | 54.590 | 54.934 | 90.491 | 89.954 | 90.222 | 1178.13 | 776 |
| Q0A2 | 52.648 | 53.039 | 85.579 | 92.030 | 88.687 | 305.39 | 878 |
| Q0A3 | 54.197 | 54.579 | 86.671 | 92.260 | 89.379 | 276.85 | 877 |
| Q3A0 | 53.337 | 53.680 | 89.082 | 89.706 | 89.393 | 107.25 | 226 |
| Q3A1 | 51.837 | 52.171 | 88.192 | 89.724 | 88.951 | 55.29 | 192 |
| Q3A2 | 51.972 | 52.307 | 84.988 | 91.587 | 88.164 | 16.92 | 169 |
| Q3A3 | 53.005 | 53.333 | 85.724 | 91.766 | 88.642 | 16.05 | 164 |
| MP | 18.348 | 20.173 | 71.249 | 81.492 | 76.027 | 64.71 | 876 |
| ***A. thaliana* vs. *G. max* (AG6)** | | | | | | | |
| Method | GN (%) | GP (%) | EN (%) | EP (%) | EF (%) | WCT (s) | MEM (MB) |
| Q0A0 | 72.736 | 74.174 | 89.542 | 89.430 | 89.486 | 142.88 | 1127 |
| Q0A1 | 71.266 | 72.379 | 88.465 | 89.429 | 88.944 | 75.41 | 726 |
| Q0A2 | 69.629 | 71.054 | 86.004 | 91.287 | 88.567 | 17.58 | 524 |
| Q0A3 | 69.128 | 70.422 | 86.051 | 90.837 | 88.379 | 15.82 | 522 |
| Q3A0 | 69.161 | 70.648 | 87.215 | 88.046 | 87.628 | 13.02 | 74 |
| Q3A1 | 68.660 | 70.137 | 86.845 | 88.573 | 87.700 | 7.60 | 57 |
| Q3A2 | 67.591 | 69.021 | 85.021 | 90.071 | 87.473 | 2.81 | 72 |
| Q3A3 | 67.324 | 68.724 | 85.045 | 89.744 | 87.331 | 2.67 | 72 |
| MP | 16.071 | 17.894 | 55.724 | 67.702 | 61.132 | 14.91 | 298 |
| ***D. melanogaster* vs. *M. domestica* (DM7)** | | | | | | | |
| Method | GN (%) | GP (%) | EN (%) | EP (%) | EF (%) | WCT (s) | MEM (MB) |
| Q0A0 | 60.996 | 61.793 | 85.979 | 79.357 | 82.535 | 718.60 | 2019 |
| Q0A1 | 60.267 | 60.989 | 85.370 | 79.572 | 82.369 | 374.15 | 776 |
| Q0A2 | 58.780 | 59.695 | 80.978 | 85.292 | 83.079 | 95.83 | 878 |
| Q0A3 | 57.839 | 58.676 | 80.907 | 83.909 | 82.381 | 87.80 | 877 |
| Q3A0 | 54.318 | 55.223 | 79.394 | 79.490 | 79.442 | 7.02 | 226 |
| Q3A1 | 55.638 | 56.565 | 81.190 | 80.733 | 80.961 | 5.57 | 192 |
| Q3A2 | 54.955 | 55.871 | 78.317 | 84.337 | 81.216 | 4.45 | 169 |
| Q3A3 | 54.121 | 54.997 | 78.091 | 83.270 | 80.597 | 4.52 | 164 |
| MP | 14.403 | 15.899 | 50.184 | 61.843 | 55.407 | 78.98 | 876 |
| ***A. thaliana* vs. *Z. mays* (AZ8)** | | | | | | | |
| Method | GN (%) | GP (%) | EN (%) | EP (%) | EF (%) | WCT (s) | MEM (MB) |
| Q0A0 | 87.000 | 87.147 | 95.853 | 95.013 | 95.431 | 428.38 | 1152 |
| Q0A1 | 86.545 | 86.665 | 95.562 | 94.924 | 95.242 | 223.36 | 1065 |
| Q0A2 | 82.020 | 82.183 | 93.065 | 94.481 | 93.767 | 52.19 | 570 |
| Q0A3 | 81.921 | 82.026 | 93.197 | 94.300 | 93.745 | 47.62 | 569 |
| Q3A0 | 85.743 | 85.870 | 95.278 | 94.700 | 94.988 | 35.48 | 76 |
| Q3A1 | 85.683 | 85.811 | 95.238 | 94.754 | 94.996 | 20.37 | 63 |
| Q3A2 | 84.475 | 84.593 | 94.496 | 94.857 | 94.676 | 6.72 | 114 |
| Q3A3 | 84.396 | 84.513 | 94.494 | 94.793 | 94.643 | 6.36 | 114 |
| MP | 55.475 | 55.796 | 75.871 | 84.080 | 79.765 | 64.23 | 93 |
| ***D. melanogaster* vs. *A. gambiae* (DA9)** | | | | | | | |
| Method | GN (%) | GP (%) | EN (%) | EP (%) | EF (%) | WCT (s) | MEM (MB) |
| Q0A0 | 45.829 | 47.060 | 74.715 | 70.990 | 72.805 | 437.12 | 2167 |
| Q0A1 | 44.943 | 46.082 | 74.068 | 71.698 | 72.864 | 314.18 | 751 |
| Q0A2 | 43.399 | 44.659 | 67.216 | 76.916 | 71.740 | 48.72 | 866 |
| Q0A3 | 42.019 | 43.202 | 67.130 | 75.246 | 70.956 | 44.44 | 865 |
| Q3A0 | 39.609 | 40.793 | 66.981 | 70.391 | 68.644 | 19.67 | 302 |
| Q3A1 | 40.330 | 41.536 | 68.596 | 72.277 | 70.389 | 11.71 | 298 |
| Q3A2 | 38.888 | 40.076 | 63.455 | 74.866 | 68.690 | 4.07 | 483 |
| Q3A3 | 38.043 | 39.181 | 63.388 | 73.683 | 68.149 | 3.92 | 471 |
| MP | 6.200 | 7.578 | 34.681 | 50.080 | 40.982 | 40.10 | 340 |

A0–3: Calculation mode of Spaln3

V2: Spaln2

MP: Miniprot

GN: Gene-level sensitivity

GP: Gene-level specificity

EN: Exon-level sensitivity

EP: Exon-level specificity

EF: Exon-level F1 measure

WCT: Wall clock time

MEM: Maximum resident memory

**
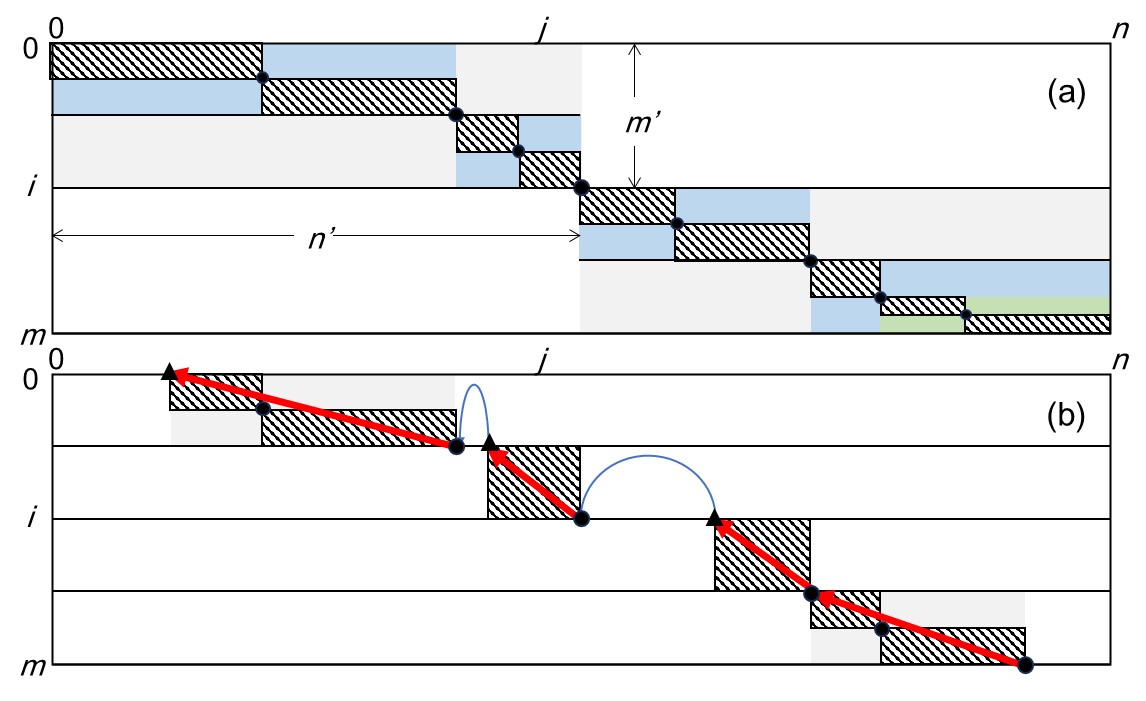
**

**Figure S1. Recursive single-intermediate unidirectional Hirschberg (SIUDH) method (a) and multi-intermediate unidirectional Hirschberg (MIUDH) method (b).** (a) Recalculation areas at the first (gray), second (blue), and third (green) recursions are shown by a colored background. The lengths of query and genomic segment subsequences subjected to the first recursion are indicated by *m’* and *n’*, respectively. (b) Vertical and horizontal links are represented by red arrows and blue arcs, respectively. Hatched boxes in (a) and (b) indicate areas where the ordinary traceback algorithm is applied.

// initialization

$\text{for}\text{ }j\in\left[ 0,r^{\text{U}} \right]\text{ }\text{\{}$ // $i=0; r=j-3*i=j$

$$H_{r}=\text{MAX}\left[ {\sigma_{j-1}^{\text{I}},0,H}_{r-1}+w\left( 1 \right),H_{r-2}+w\left( 2 \right),H_{r-3}+w\left( 3 \right)+\sigma_{j-1}^{\text{E}},H_{r-3}^{1}-u+\sigma_{j-1}^{\text{E}} \right]$$

$$X_{r}=\text{SEL}\left[ j-3,j,j-1,j-2,j-3,X_{r-3} \right]$$

}

$\text{for}\text{ }c\in\left[ 0,k \right]\text{ }\text{\{}$ // intermediates

$ii\left[ c \right]=c*m/k$

$$\text{for}\text{ }d\in\left[ 0,1 \right] \left\{ \text{for}\text{ }j\in\left[ 0,n \right] \left\{ VL\left[ c \right]\left[ d \right]\left[ j \right]=HL\left[ c \right]\left[ d \right]\left[ j \right]=\$ \right\} \right\}$$

}

*x* // recurrence formula

Alignment path

Intron

*X_r_=j*

*X_r_ =X^2^_r_=j*

*h*

*HL*[*c*][0][*j*]= *h*

*j*

*X_r_=j*

*j*

*j*

(a)

(b)

(c)

*h*

*HL*[*c*][0][*j*]=*h*

*j*

*X_r_=j*

*h*

*j*

*HL*[*c*][1][*j*]=*h*

*X_r_= X^2^_r_= j*

(d)

(e)

$$c=0$$

$$\text{for}\text{ }i\in\left[ 1,m \right]\text{ }\text{\{}$$

$$\text{ }\text{for}\text{ }j\in\left[ \text{Max}\left[ 0,{3*i+r}^{\text{L}} \right],\text{Min}\left[ n,3*i+r^{\text{U}} \right] \right]\text{ \{}$$

$$r=j-3*i$$

$$\tau=\left( i==m \&\&\sigma_{j-1}^{\text{T}}>0 \right)?\sigma_{j-1}^{\text{T}}:0$$

$$H_{r}^{1}=\text{MAX}\left[ H_{r-1}+w\left( 1 \right),H_{r-2}+w\left( 2 \right),H_{r-3}+\left( \tau?\tau:\sigma_{j-1}^{\text{E}}+w(3) \right),H_{r-3}^{1}+\left( \tau?\tau:\sigma_{j-1}^{\text{E}}-u \right) \right]$$

$$X_{r}^{1}=\text{SEL}\left[ X_{r-1},X_{r-2},X_{r-3},X_{r-3}^{1} \right]$$

$$Y_{r}^{1}=\text{SEL}\left[ j-1,j-2,j-3,Y_{r-3}^{1} \right]; Y_{r}^{0}=Y_{r}^{2}=\$;$$

$$H_{r}^{2}=\text{MAX}\left[ H_{r+1}+w\left( 1 \right),H_{r+2}+w\left( 2 \right),H_{r+3}+w\left( 3 \right),H_{r+3}^{2}-u \right]$$

$$X_{r}^{2}=\text{SEL}\left[ X_{r+1},X_{r+2},X_{r+3},X_{r+3}^{2} \right]$$

$$H_{r}^{0}=H_{r}+S\left( a_{i-1},b_{j-1} \right)+\sigma_{j-1}^{\text{E}}$$

$$\text{for}\text{ }\text{α∈}\left[ 0,2 \right] z^{\alpha}=\mathbf{false}$$

$$\text{if}\text{ }\left( j-\delta\text{ is potential acceptor site} \right) \{$$

$$\text{for}\text{ }\alpha\in\left[ 0,2 \right] \{$$

$$h={\text{ARG}\text{MAX}}_{k\in Q^{\delta}}\left[ H_{k-3*i}^{\alpha}+\sigma_{k-\delta}^{5^{'}}+\sigma_{j-\delta}^{3^{'}}+\gamma\left( j-k \right) \right]$$

$$H_{r}^{\alpha}=\text{MAX}\left( H_{r}^{\alpha},H_{h-3*i}^{\alpha}+\sigma_{h-\delta}^{5^{'}}+\sigma_{j-\delta}^{3^{'}}+\gamma\left( j-h \right) \right)$$

$$X_{r}^{\alpha}=\text{SEL}\left[ X_{r}^{\alpha},X_{h-3*i}^{\alpha} \right]$$

$$Y_{r}^{\alpha}=\text{SEL}\left[ Y_{r}^{\alpha},h \right]$$

$$z^{\alpha}=\text{SEL}\left[ \text{false}, \text{true} \right]$$

$$\text{\}}$$

}

$$H_{r}\text{=MAX}\left[ H_{r}^{0},H_{r}^{1},H_{r}^{2} \right]\text{}$$

$$X_{r}\text{=SEL}\left[ X_{r}^{0},X_{r}^{1},X_{r}^{2} \right]$$

$$Y_{r}\text{=SEL}\left[ Y_{r}^{0},Y_{r}^{1},Y_{r}^{2} \right]$$

$$z=\text{SEL}\left[ z^{0},z^{1},z^{2} \right]$$

$$\text{if}\text{ }\left( j-\delta\text{ is potential }\text{donor}\text{ site} \right)$$

$$\text{for }\text{α∈}\left[ 0,2 \right]$$

$$Q^{\delta}.\text{push}\left( H_{r}^{\alpha}+\sigma_{h-\delta}^{5^{'}},j,\alpha\right)$$

$\text{if}\text{ }\left( i==ii\left[ c \right] \right)\text{ }\text{\{}$ // intermediate

$$VL\left[ c \right]\left[ 0 \right]\left[ j \right]=X_{r}\%n$$

$$VL[c]\left[ X_{r}/n \right]\left[ j \right]=X_{r}\%n$$

$$X_{r}=j; X_{r}^{2}=j+n$$

$$HL\left[ c \right]\left[ 0 \right]\left[ j \right]=Y_{r}$$

$$HL\left[ c \right]\left[ 1 \right]\left[ j \right]=Y_{r}^{2}$$

$$\text{if}\text{ }\left( z \right) Y_{r}=j$$

}

$\text{ }$}

$$\text{if} \left( i==ii\left[ c \right] \right)\text{ ++}c$$

}

// traceback

$$j=\text{ARGMAX}_{r\in\left[ r^{\text{L}},n-3*m \right]}\left[ H_{r} \right]$$

$$d=0$$

$$\text{for}\text{ }\left( c=k;\text{--}c\geq0;\text{ } \right) \{$$

$$l=X_{r}\%n; e=X_{r}/n$$

$$\text{Align}\left( \boldsymbol{a}\left[ ii\left[ c \right],ii\left[ c+1 \right] \right],\boldsymbol{b}\left[ l,j \right],d,e \right)$$

$$\text{for}\text{ }\left( j=l;\left( l=HL\left[ c \right]\left[ d \right]\left[ j \right] \right)\neq\$;j=l \right)$$

$$\text{Output}\left( ii\left[ c \right],j \right)$$

$$d=e$$

}

**Figure S2. Pseudocode of banded DP with MIUDH algorithm.** $r^{\text{L}}$ and $r^{\text{U}}$ are the lower and upper bounds within which the banded DP is calculated. $H_{r}$ and $H_{r}^{\alpha}$ denote alignment scores. $X_{r}$ and $X_{r}^{\alpha}$ denote the *j*-coordinates at which the alignment leading to the current position crosses the latest intermediate. $Y_{r}$ denotes the nearest acceptor site on the horizon or the start site of the current horizontal gap, or the donor site of the current intron. Boolean variable *z* indicates whether *j* is a candidate acceptor site or not. The superscript $\alpha$=0, 1, or 2 indicates diagonal, horizontal, or vertical moves, respectively. Undefined score variables, such as $H_{r}$ for $r\in\left[ r^{\text{L}},-1 \right]$ in the initialization process, are assigned to $-\infty$. $VL\left[ c \right]\left[ d \right]\left[ j \right]$ and $HL\left[ c \right]\left[ d \right]\left[ j \right]$ are vertical and horizontal links, respectively, and $ marks the end of a link. Several typical cases are depicted in the inset. Coding potential, $\sigma_{j}^{\text{E}}$, translational start signal, $\sigma_{j}^{\text{I}}$, and translational stop signal, $\sigma_{j}^{\text{T}}$, are assigned to the codon center, whereas splicing donor signal, $\sigma_{j}^{5^{'}}$, and acceptor signal, $\sigma_{j}^{3^{'}}$, are assigned to the splicing boundary at which the phase $\delta=0$. Genomic sequence ***b*** is converted into tron code (Gotoh, 2000) in advance. $S\left( a,b \right)$ is the similarity measure between residues *a* and *b*, -*u* is the gap extension penalty, and $w\left( k \right)$ is the gap penalty of length *k* bp that includes gap-open, gap-extension, and frame-shift penalties. $\gamma\left( k \right)$ is the intron penalty of length *k*. Operators MAX and following SEL(s) are synchronized so that if the *k*-th term is selected by the MAX operation, the *k*-th term within the square brackets is selected by the following SEL operator(s). $Q^{\delta} \left( \delta\in\left[ -1,1 \right] \right)$ denotes a priory queue of the key of $H_{r}^{\alpha}+\sigma_{j-\delta}^{5^{'}}$, which stores information of the splicing donor site provided by the ‘push’ function. Function $\text{Align}\left( \boldsymbol{a}\left[ p,q \right],\boldsymbol{b}\left[ l,r \right],d,e \right)$ performs traceback-based spliced alignment between substrings $\boldsymbol{a}\left[ p,q \right]$ and $\boldsymbol{b}\left[ l,r \right]$, where arguments *d* and *e* negatively control whether the terminal gap-open penalty is imposed or not. Function $\text{Output}\left( i,j \right)$ outputs a coordinate pair $\left( i,j \right)$ that participates in the optimal path of the spliced alignment.

Query amino acid sequence

Genomic DNA sequence

*N_R_*

3*N*_R_

**Figure S3. Anti-rhombic coordinate system used for SIMD-based banded DP calculation.** Blue slanted lines indicate vector registers used for filling the current register (red slanted line) that processes *N*_R_ (= 4 in this example) rows in parallel. The brown horizontal line indicates an initialized array of *n* + 3*m* size. The orange horizontal line indicates the part of the array already refreshed in the current iteration cycle, and the green horizontal line indicates the part of the array to be used in the current iteration cycle. The light green arrow indicates a push-pull queue used for efficiently accessing sparse locations of potential donor and accepter sites indicated by blue and red vertical lines, respectively.


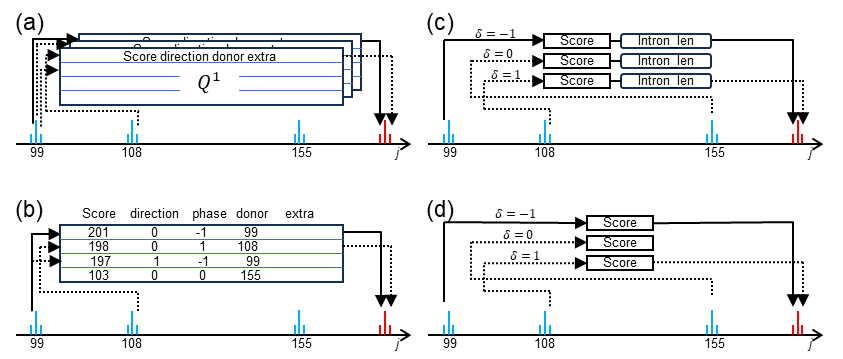


**Fig. S4. Differences among Modes A0-A3 in calculating intron penalty.** In Modes A1-A3, *N*_R_ consecutive rows are processed in parallel with vector registers. Potential donor sites and acceptor sites are indicated by vertical blue and red lines, respectively. (a) Mode A0. For each phase $\delta\in\left[ -1,1 \right]$ of a potential donor site at coordinate *j*, we make a record *R*_0_ consisting of four fields, ‘score’, ‘direction’, ‘coordinate’, and ‘extra’. The ‘score’ is the alignment score of the ‘direction’ (diagonal: 0, horizontal: 1, and vertical: 2) at the current cell + 5’ splicing signal strength. The fourth field is used differently in traceback-based and UDH methods, the details of which are not described here. For each phase, we also prepare a priority queue $Q^{\delta}$ storing maximally *Q*_S0_ (4 by default) *R*_0_ records. Each priority queue is updated or unchanged at subsequent potential donor sites so as to maintain the sorted order of the score values. At each potential acceptor site of phase $\delta$, spliced alignment scores are obtained from the records in $Q^{\delta}$ and the 3’ splicing signal strength. If the maximum spliced score for a particular direction is greater than the corresponding current alignment score, the current score and associated variables are replaced by the spliced score and its associates. (b) Mode A1. For each potential donor site at $\delta=1$, splicing-related operations of the three phases are collectively processed. We make a single record *R*_1_ consisting of five fields, ‘score’, ‘direction’, ‘phase’, ‘coordinate’, and ‘extra’. We also prepare a priority queue storing maximally *Q*_S1_ (4 by default) *R*_1_ recodes. Potential donor (or acceptor) sites within a genomic segment of 3*N*_R_ bp long are efficiently located with a push-pull queue (Fig. S3). Other procedures are the same as those of Mode A0. (c) Mode A2. For each phase of a donor site, ‘score’ is obtained as described above. If the score is greater than the value of a variable $S^{\delta}$ (initialized as $-\infty$), $S^{\delta}$ is replaced by the new score and the intron length $L^{\delta}$is reset to 0. In all the other cases, $L^{\delta}$is incremented by one at each *j*. At a potential acceptor site of phase $\delta$, a spliced alignment score is obtained as $S^{\delta}$+ 3’ splicing signal strength + intron penalty $\gamma\left( L^{\delta} \right)$ of length $L^{\delta}$. If the spliced alignment score is greater than the current alignment score, the current score is replaced by the spliced score. If we use a step function (Fig. S5) as the intron penalty function, we can obtain $\gamma\left( L^{\delta} \right)$s of *N*_R_ rows in parallel with O(*N*_S_) operations, where *N*_S_ is the number of steps of the step function. (d) Mode A3. This simplest mode is nearly identical to Mode A2. However, as the intron penalty is independent of intron length, $L^{\delta}$is dispensable, requiring only simple calculation of a spliced alignment score. In Modes A2/A3, a gap that strides across an intron may not be rigorously penalized as the directional information is lost. Moreover, split codons are neglected for calculating alignment score.

**
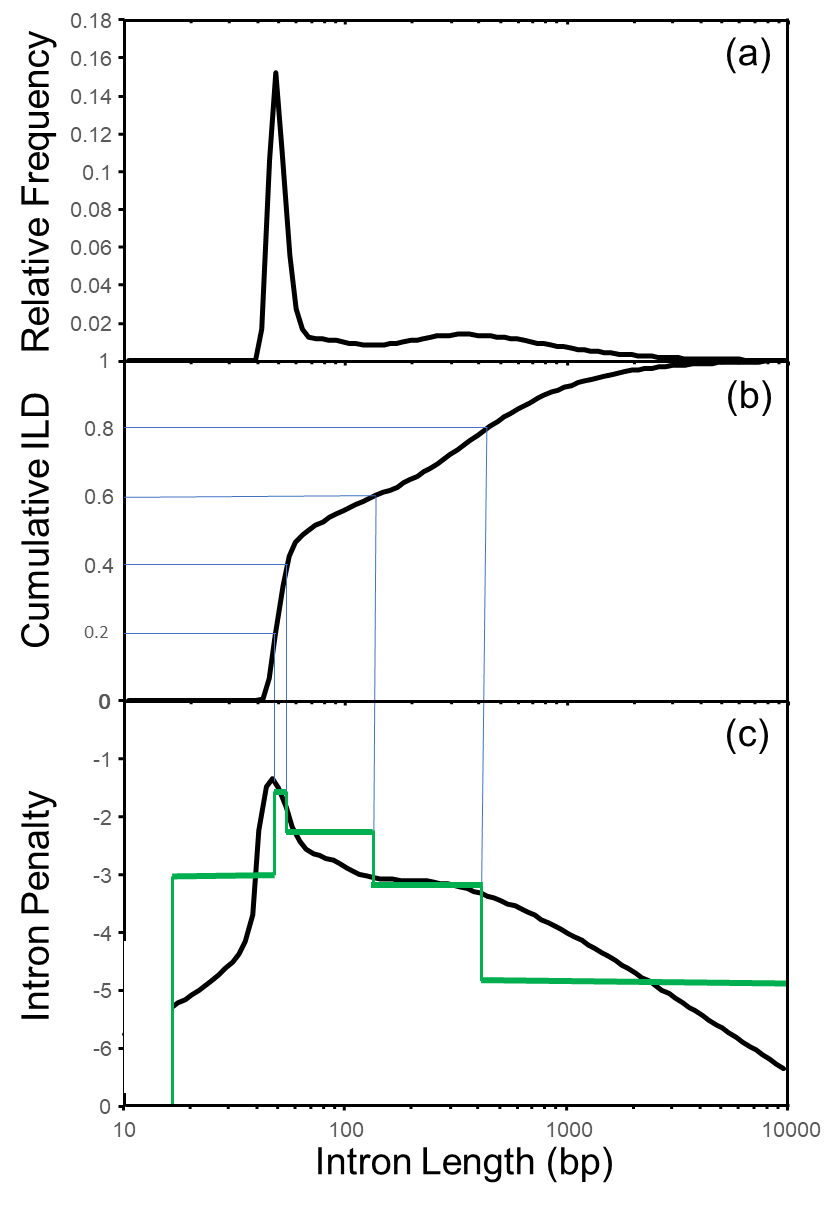
**

**Figure S5. Coarse-grained intron penalty.** (a) Intron length distribution (ILD) of *C. elegans*. (b) Cumulative ILD. (c) Intron penalty (black solid line) and its approximation by step function (green solid line). Each step is delineated by equally divided quantile points (thin lines). The value of each step is defined as the mean of the intron penalties within the interval.

**
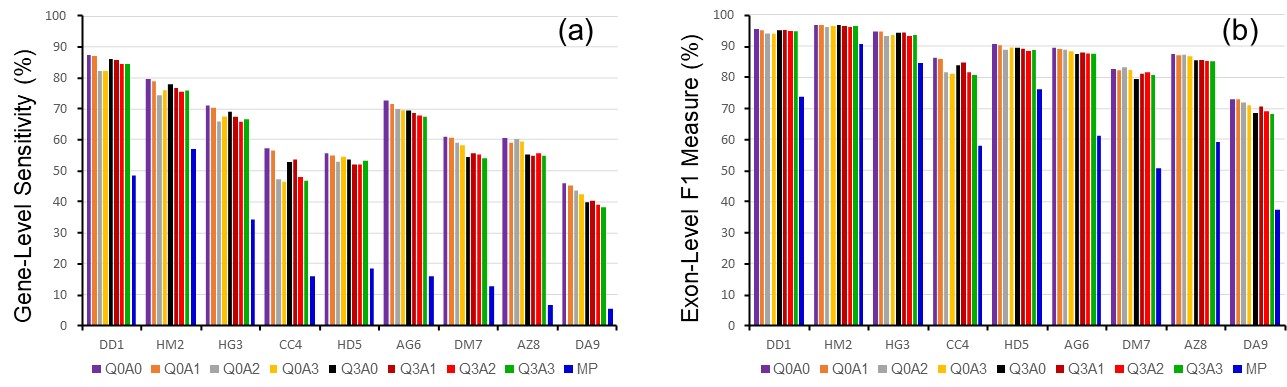
**

**Figure S6. Mean accuracies of spliced alignment of nine pairs of genomes and queries.** (a) Gene-level sensitivity. (b) Exon-level F1 measure. The genome and query pairs are represented by three-letter codes and arranged in descending order of mean amino acid identifiers, as shown in Table 1. Q0: banded DP algorithm, Q3: heuristic algorithm with three levels of HSP search. A[0–3]: DP calculation modes of Spaln3. MP: Miniprot.
